# Lipocalin-2 and neutrophil activation in pancreatic cancer cachexia

**DOI:** 10.3389/fimmu.2023.1159411

**Published:** 2023-03-15

**Authors:** Min Deng, Merel R. Aberle, Annemarie A. J. H. M. van Bijnen, Gregory van der Kroft, Kaatje Lenaerts, Ulf P. Neumann, Georg Wiltberger, Frank G. Schaap, Steven W. M. Olde Damink, Sander S. Rensen

**Affiliations:** ^1^ Department of Surgery and School of Nutrition and Translational Research in Metabolism (NUTRIM), Maastricht University, Maastricht, Netherlands; ^2^ Department of General, Visceral- and Transplantation Surgery, Rheinisch-Westfälische Technische Hochschule (RWTH) Aachen University, Aachen, Germany

**Keywords:** cancer cachexia, neutrophil activation, innate immunity, appetite, lipocalin-2, nutritional status, complement

## Abstract

**Background:**

Cancer cachexia is a multifactorial syndrome characterized by body weight loss and systemic inflammation. The characterization of the inflammatory response in patients with cachexia is still limited. Lipocalin-2, a protein abundant in neutrophils, has recently been implicated in appetite suppression in preclinical models of pancreatic cancer cachexia. We hypothesized that lipocalin-2 levels could be associated with neutrophil activation and nutritional status of pancreatic ductal adenocarcinoma (PDAC) patients.

**Methods:**

Plasma levels of neutrophil activation markers calprotectin, myeloperoxidase, elastase, and bactericidal/permeability-increasing protein (BPI) were compared between non-cachectic PDAC patients (n=13) and cachectic PDAC patients with high (≥26.9 ng/mL, *n*=34) or low (<26.9 ng/mL, *n*=34) circulating lipocalin-2 levels. Patients’ nutritional status was assessed by the patient-generated subjective global assessment (PG-SGA) and through body composition analysis using CT-scan slices at the L3 level.

**Results:**

Circulating lipocalin-2 levels did not differ between cachectic and non-cachectic PDAC patients (median 26.7 (IQR 19.7-34.8) *vs*. 24.8 (16.6-29.4) ng/mL, *p*=0.141). Cachectic patients with high systemic lipocalin-2 levels had higher concentrations of calprotectin, myeloperoxidase, and elastase than non-cachectic patients or cachectic patients with low lipocalin-2 levels (calprotectin: 542.3 (355.8-724.9) *vs*. 457.5 (213.3-606.9), *p*=0.448 *vs*. 366.5 (294.5-478.5) ng/mL, *p*=0.009; myeloperoxidase: 30.3 (22.1-37.9) *vs*. 16.3 (12.0-27.5), *p*=0.021 *vs*. 20.2 (15.0-29.2) ng/mL, *p*=0.011; elastase: 137.1 (90.8-253.2) *vs*. 97.2 (28.8-215.7), *p*=0.410 *vs*. 95.0 (72.2-113.6) ng/mL, *p*=0.006; respectively). The CRP/albumin ratio was also higher in cachectic patients with high lipocalin-2 levels (2.3 (1.3-6.0) as compared to non-cachectic patients (1.0 (0.7-4.2), *p*=0.041). Lipocalin-2 concentrations correlated with those of calprotectin (*r_s_* =0.36, *p*<0.001), myeloperoxidase (*r_s_* =0.48, *p*<0.001), elastase (*r_s_* =0.50, *p*<0.001), and BPI (*r_s_* =0.22, *p*=0.048). Whereas no significant correlations with weight loss, BMI, or L3 skeletal muscle index were observed, lipocalin-2 concentrations were associated with subcutaneous adipose tissue index (*r_s_* =-0.25, *p*=0.034). Moreover, lipocalin-2 tended to be elevated in severely malnourished patients compared with well-nourished patients (27.2 (20.3-37.2) *vs*. 19.9 (13.4-26.4) ng/mL, *p*=0.058).

**Conclusions:**

These data suggest that lipocalin-2 levels are associated with neutrophil activation in patients with pancreatic cancer cachexia and that it may contribute to their poor nutritional status.

## Introduction

Cancer cachexia is a multifactorial syndrome characterized by ongoing body weight loss that results in reduced quality of life, low tolerance for anti-cancer treatment, and poor survival (1). It is highly prevalent in many types of cancers but is most common in pancreatic cancer, where it affects up to 80% of patients with frequently more than 10% body weight loss (2). The molecular mechanisms underlying the development of cancer cachexia remain poorly defined, although tumor-derived catabolic factors such as activins, myostatin, and pro-inflammatory cytokines arising from tumor-immune system crosstalk are thought to contribute to its progression (3, 4). For example, elevated circulating TNF-α, IL-6, and GDF-15 have been reported to be associated with the severity of cachexia in cancer patients and mouse models (5, 6). Furthermore, functional data support the participation of pro-inflammatory factors in tumor progression and cachectic features such as adipose tissue lipolysis and muscle wasting (7, 8).

Neutrophils are the most abundant immune cells in the circulation of humans (up to 70% of the total white blood cell count) and form an essential part of the innate immune response against infection and various other inflammatory cues. They have also been implicated in pancreatic cancer. For instance, Pratt et al. have shown that gene signatures associated with neutrophil recruitment are increased in pancreatic ductal adenocarcinoma (PDAC) tissue as compared to normal pancreatic tissue (9). Furthermore, high levels of circulating and intratumoral neutrophils have been shown to correlate with poor survival in patients with pancreatic cancer (10). Additionally, neutrophils can promote pancreatic tumor metastasis by the formation of so-called neutrophil extracellular traps (NETs). Pancreatic cancer cells can induce the release of NETs *in vitro* (11), and NETs are elevated in the blood of mice and patients with PDAC (12, 13). In the context of cancer cachexia, emerging investigations revealed increased circulating neutrophils both in patients with cancer cachexia and in different mouse models of cancer cachexia (1, 14, 15). Furthermore, blocking C-C motif chemokine receptor 2 (CCR2) signaling by neutrophils infiltrated in the velum interpositum region of the brain has been shown to ameliorate cachexia-associated metabolic alterations in mouse models of pancreatic cancer cachexia (16).

Upon activation by inflammatory stimuli, neutrophils can secrete a plethora of cytotoxic proteins, including neutrophil elastase (NE), myeloperoxidase (MPO), calprotectin, bactericidal/permeability-increasing protein (BPI), and lipocalin 2 (LCN-2, also known as neutrophil gelatinase-associated lipocalin or NGAL). LCN-2 can also be released by other cell types including macrophages, adipocytes, and hepatocytes (17). This protein has been associated with several diseases such as obesity, type 2 diabetes, breast cancer, and pancreatic cancer (17, 18). The biological functions of LCN-2 are diverse and include antibacterial, anti-inflammatory, as well as pro-metastatic actions (17). Recently, LCN-2 was identified as a bone-derived hormone with central metabolic regulatory effects which suppresses appetite by binding to the melanocortin 4 receptor (MC4R) (19). Furthermore, a study in a mouse model of pancreatic cancer cachexia revealed that circulating LCN-2 levels were increased in cachectic mice and correlated with anorexia and muscle loss; genetic deletion of LCN-2 ameliorated cachexia-associated anorexia (20). In the same study, a significant increase in LCN-2 mRNA was found in circulating neutrophils of cachectic mice and it was suggested that together with the bone marrow compartment, neutrophils are the predominant source of circulating LCN-2 during cancer cachexia development. Using IL6- and Myd88- knockout mice, it was shown that LCN-2 is an inflammation-induced factor in cancer cachexia (20). Although it is clear that neutrophil and bone marrow derived-LCN-2 contributes to cancer cachexia development by suppressing appetite in mice, whether the same mechanism applies in PDAC patients with cachexia remains unknown.

Given that systemic inflammation is a hallmark of cancer cachexia, and since neutrophils release cytotoxic proteins and LCN-2 upon activation by inflammatory stimuli, we hypothesized that neutrophils contribute to systemic inflammation and the release of LCN-2 in cachectic patients with pancreatic cancer. We aimed to 1) investigate the association between circulating levels of LCN-2 and neutrophil activation markers as well as features of cachexia in PDAC patients; 2) determine whether there is a link between LCN-2 levels and appetite in pancreatic cancer patients with cachexia.

## Materials and methods

### Patients

81 patients undergoing pancreaticoduodenectomy for suspected adenocarcinoma of the pancreas at the Maastricht University Medical Centre (MUMC+) or University Hospital RWTH Aachen were enrolled in this study. Exclusion criteria were neoadjuvant chemo- and/or radiotherapy and the presence of another malignancy. This study was approved by the Medical Ethical Board of the MUMC+ in line with the ethical guidelines of the 1975 Declaration of Helsinki, and written informed consent was obtained from each subject (METC 13-4-107 and 2019-0977 for patients from MUMC+, EK 172/17 for patients from Uniklinik Aachen).

### Diagnosis of cancer cachexia and screening of cachexia status

Cachexia was defined according to the international consensus definition as 1) weight loss >5% over the past 6 months in the absence of starvation, and/or 2) BMI <20 kg/m^2^ and >2% ongoing weight loss, and/or 3) sarcopenia and >2% ongoing weight loss. Patients were diagnosed with cancer cachexia if ≥1 of the criteria were met (1). Body weight loss was reported by the patient and body weight data were retrieved from the medical record. Sarcopenia was defined using established cut-offs in patients with pancreatic cancer as L3-skeletal muscle index (L3-SMI) <45.1 cm^2^/m^2^ in males and <36.9 cm^2^/m^2^ in females (21).

Body composition parameters were quantified by analyzing a cross-sectional CT image at the third lumbar (L3) vertebra that was acquired preoperatively for diagnostic purposes, using sliceOmatic 5.0 software (TomoVision, Magog, Canada) for Windows. Using predefined Hounsfield Unit (HU) ranges, the total cross-sectional area (cm^2^) of skeletal muscle (SM) tissue (-29 to 150 HU) was determined. In addition, the total areas of visceral adipose tissue (VAT, -150 to -50 HU) and subcutaneous adipose tissue (SAT) as well as intramuscular adipose tissue (IMAT) (-190 to -30 HU) were assessed. Tissue areas (cm^2^) were adjusted for patient height to calculate the respective L3-indices (L3-SMI, L3-VATI, L3-SATI) in cm^2^/m^2^, which correspond well with total body muscle and adipose tissue mass (22). Previously published validated sex-specific cut-off values (SMI, 45.1 cm^2^/m^2^ for men and 36.9 cm^2^/m^2^ for women) that were established from a local MUMC+ cohort including pancreatic cancer patients (21) were used for the CT-derived body composition analysis. In addition, the skeletal muscle radiation attenuation (SMRA), which reflects the extent of lipid accumulation in the muscle, was calculated as the average HU value of the total tissue area for muscle (i.e. within the specified range of -29 to 150 HU). A total of 80 patients were included for body composition analysis (one patient had no CT-scan available). L3-SATI could not be accurately assessed in 8 patients because of incomplete CT-scans not showing all tissue.

### Assessment of patient’s nutritional status and appetite

Patients’ nutritional status was assessed by using the patient-generated subjective global assessment, a validated nutritional screening tool (23) (PG-SGA, category A: well-nourished, category B: moderate malnutrition, category C: severe malnutrition). Patient’s appetite was assessed according to the question in box 2 of the PG-SGA questionnaire and rated as normal food intake (unchanged or more than usual) or less than usual food intake.

### Plasma preparation

A venous blood sample was obtained preoperatively for measuring clinical laboratory data and levels of neutrophil activation markers. To avoid artefactual neutrophil activation during plasma preparation, venous blood was collected in EDTA tubes and gently centrifuged at 1500xg at 4°C for 15 min without brake, after which plasma aliquots were stored at -80°C until analysis.

### ELISAs

Levels of circulating neutrophil activation markers calprotectin, myeloperoxidase (MPO), elastase, bactericidal permeability increasing protein (BPI), and LCN-2 were measured by solid-phase enzyme-linked immunosorbent assays (ELISA) based on the sandwich principle, according to the manufacturer’s instructions (Hycult Biotech, Uden, The Netherlands; Human calprotectin, Catalog #HK379; Human MPO, Catalog # HK324; Human elastase, Catalog # HK319; Human BPI, Catalog # HK314; Human LCN-2, Catalog # HK330). All plasma samples were analyzed in duplicate in the same run. The intra- and inter-assay coefficients of variance of the various assays were <10%. Clinical laboratory data including circulating C-reactive protein (CRP), albumin, neutrophils (%), and lymphocytes (%) were measured in the clinical setting. For some of the patients, these clinical data were not available, the exact number of the studied patients for these data is indicated in each figure legend.

### Statistical analysis

Statistical analysis was performed using Prism 7.0 for Windows (GraphPad Software Inc., San Diego, CA) and R (R-4.2.0 for Windows). Data are presented as the median with interquartile range (IQR). Non-parametric tests were used for statistical analysis (Mann-Whitney U test for analysis of two groups; Kruskal-Wallis test followed by Dunn’s post-testing for analysis of multiple groups). Correlations were calculated using Spearman’s correlation coefficient (*r_s_*), and Spearman’s correlation matrix was generated by the Corrplot R package (24). *P* values <0.05 were considered statistically significant.

## Results

### Characteristics of the study cohort

A total of 81 patients with PDAC were enrolled in this study (31 females and 50 males). The median age of patients was 69.0 years. CT scan-based body composition analysis showed that 63.7% (n=51) of patients were sarcopenic, with a median L3-SMI of 47.5 (42.8-51.2) cm^2^/m^2^ for males and 37.5 (35.1-40.5) cm^2^/m^2^ for females. The median L3-VAT and L3-SAT indices were 40.5 (25.3-74.5) cm^2^/m^2^ and 46.7 (34.7-58.5) cm^2^/m^2^.

**Figure 1:**
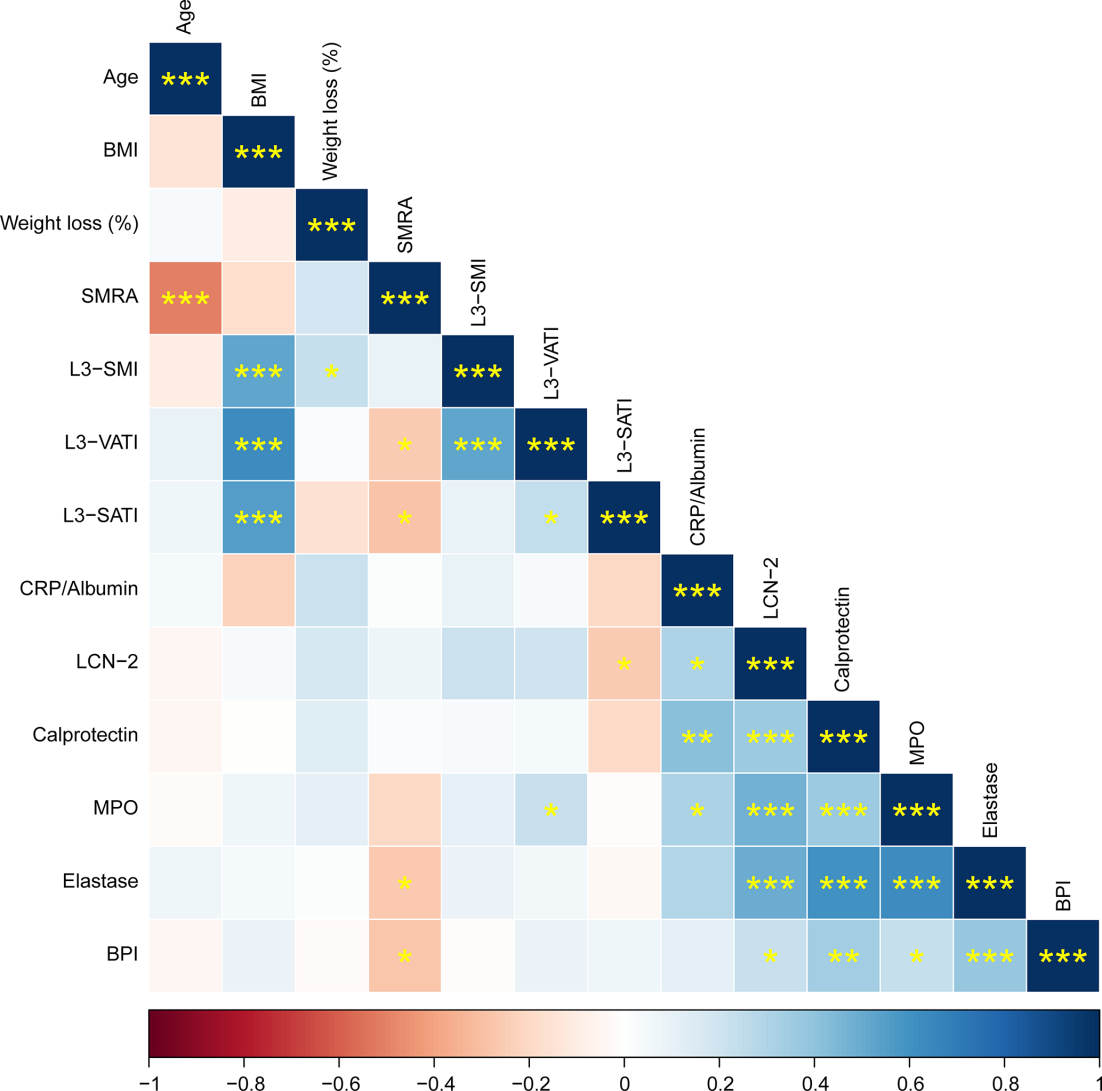
Correlation matrix showing Spearman correlations between patient characteristics and circulating factors. Positive correlations are shown in blue, negative correlations in red. The color intensity indicates the Spearman’s correlation coefficient (bottom legend). The asterisks indicate the level of statistical significance (*p < 0.05, **p < 0.01, ***p < 0.001).

Given that LCN-2 levels have been reported to correlate with fat and lean mass wasting (two key features of cachexia) in patients with pancreatic cancer (20), we subdivided cachectic patients into groups with high or low LCN-2 using a median cut-off value of 26.9 ng/mL (see **Table 1**). The median weight loss of the non-cachectic patients was 3.1 (0.7-3.6) %, which was significantly less than the weight loss of the cachectic patients with high LCN-2 (median 11.5 (7.8-14.1) %, *p*<0.001) and the cachectic patients with low LCN-2 (median 8.4 (6.5-14.2) %, *p*<0.001). According to the PG-SGA, 95% (n=18) of patients with cachexia and high LCN-2 were malnourished (42% moderate malnutrition (category B) + 53% severe malnutrition (category C)), which was higher than the prevalence of malnutrition in patients without cachexia (50%; 40% category B + 10% category C) and in patients with cachexia with low LCN-2 (74%; 53% category B + 21% category C). Further patient characteristics are presented in **Table 1**, and Spearman correlations between studied variables are shown in **Figure 1**.

**Table 1:** General characteristics of included patients.

	Overall	No cachexia	Cachexia	Cachexia	p-value*
			with low LCN-2	with high LCN-2	
*n*	81	13	34	34	
Age (years)	69.0 (62.0, 75.0)	67.0 (58.0, 72.0)	71.9 (64.1, 75.8)	68.7 (61.5, 75.9)	0.463
Sex = M/F (%)	50/31 (61.7/38.3)	7/6 (53.8/46.2)	17/17 (50.0/50.0)	26/8 (76.5/23.5)	0.066
BMI (kg/m^2^)	23.8 (21.9, 26.5)	25.0 (23.5, 26.2)	23.0 (21.7, 25.9)	24.0 (22.0, 26.7)	0.327
Weight loss percentage (%)	8.3 (5.2, 13.9)	3.1 (0.7, 3.6)	8.4 (6.5, 14.2)*	11.5 (7.8, 14.1)*	***<*0.001**
Sarcopenia = Yes/No (%)	51/29 (63.7/36.2)	9/4 (69.2/30.8)	20/13 (60.6/39.4)	22/12 (64.7/35.3)	0.863
PG-SGA *n* (%)					**0.029**
A	11 (22.9)	5 (50.0)	5 (26.3)	1 (5.3)	
B	22 (45.8)	4 (40.0)	10 (52.6)	8 (42.1)	
C	15 (31.2)	1 (10.0)	4 (21.1)	10 (52.6)	
Normal/Less food intake (%)	24/38 (38.7/61.3)	7/6 (53.8/46.2)	8/17 (32.0/68.0)	9/15 (37.5/62.5)	0.448
SMRA (HU)	35.4 (30.4, 42.8)	37.5 (35.3, 38.9)	33.7 (28.6, 43.5)	35.4 (30.3, 41.5)	0.664
Male	36.2 (33.1, 43.2)	36.6 (35.9, 38.9)	34.9 (32.5, 44.0)	36.1 (31.7, 42.9)	0.787
Female	30.6 (27.5, 39.2)	37.5 (26.7, 38.5)	30.6 (27.4, 42.6)	30.0 (29.0, 35.5)	0.806
IMAT (cm^2^)	7.9 (4.3, 14.0)	7.1 (3.9, 12.5)	7.7 (4.5, 13.2)	8.9 (4.6, 15.0)	0.782
Male	7.6 (4.2, 13.9)	4.6 (3.9, 9.4)	6.3 (4.4, 12.6)	9.0 (4.1, 15.5)	0.516
Female	8.4 (6.1, 13.7)	10.7 (5.1, 13.7)	8.8 (6.1, 11.9)	7.2 (6.5, 11.1)	0.942
L3-SMI (cm^2^/m^2^)	43.3 (38.0, 49.2)	42.9 (37.2, 47.2)	42.5 (38.0, 46.2)	46.2 (39.4, 51.1)	0.173
Male	47.5 (42.8, 51.2)	47.2 (45.0, 48.5)	46.5 (42.2, 50.7)	48.0 (44.0, 51.4)	0.857
Female	37.5 (35.1, 40.5)	37.2 (36.9, 37.3)	38.9 (34.9, 42.5)	38.2 (34.5, 39.7)	0.750
L3-VATI (cm^2^/m^2^)	40.5 (25.3, 74.5)	43.5 (25.4, 50.0)	35.4 (25.0, 63.8)	51.0 (26.1, 85.2)	0.291
Male	59.7 (32.0, 87.4)	50.0 (35.2, 74.3)	49.8 (29.7, 73.9)	66.3 (36.9, 91.2)	0.436
Female	26.4 (19.6, 39.1)	28.9 (22.2, 40.7)	33.1 (20.6, 37.2)	23.0 (16.7, 29.9)	0.662
L3-SATI (cm^2^/m^2^)	46.7 (34.7, 58.5)	55.0 (39.8, 87.1)	48.8 (41.9, 67.3)	41.4 (31.5, 51.7)	0.065
Male	42.5 (32.1, 54.5)	39.8 (27.4, 52.6)	51.9 (46.2, 67.3)	40.6 (30.1, 45.5)	0.071
Female	49.7 (42.3, 81.5)	87.2 (67.7, 88.1)	47.4 (40.8, 65.7)	47.6 (42.0, 58.7)	0.093
CRP/Albumin ratio	1.6 (0.7, 4.2)	1.0 (0.4, 1.4)	1.2 (0.3, 6.2)	2.3 (1.3, 6.0)*	**0.043**

The data are presented as median + IQR. Groups were compared using the Kruskal–Wallis test. * Significant difference in comparison to the no cachexia group. BMI, body mass index; PG-SGA, patient-generated subjective global assessment; HU, Hounsfield unit; L3-IMAT, L3-intermuscular adipose tissue; SMRA, skeletal muscle radiation attenuation; L3-SMI, L3-muscle index; L3-VATI, L3-visceral adipose tissue index; L3-SATI, L3-subcutaneous adipose tissue index; CRP, C-reactive protein. The bold values means p < 0.05.

### Circulating LCN-2 is higher in males and correlates with systemic inflammation

**Figure 2:**
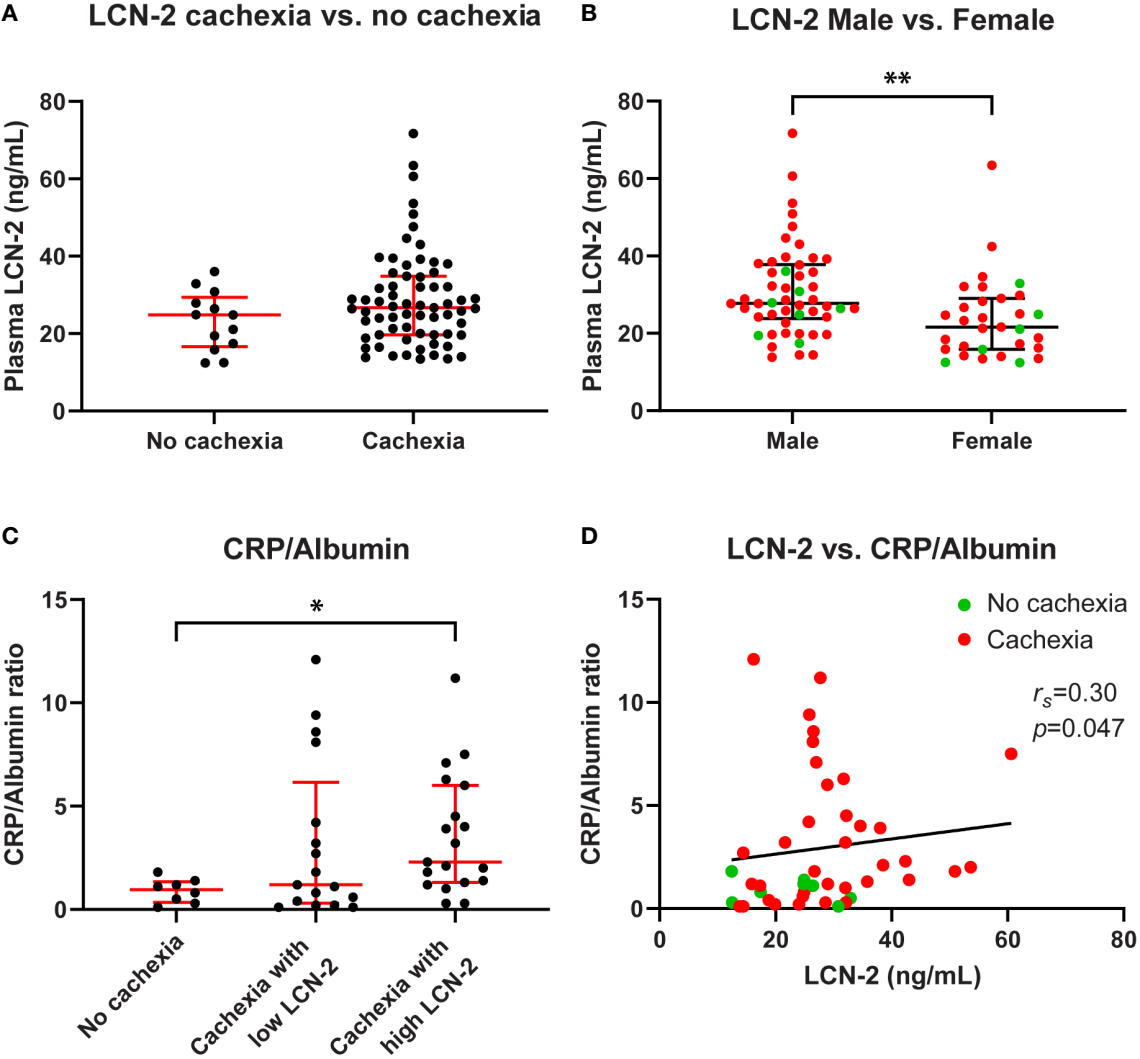
Circulating LCN-2 levels of PDAC patients differ according to sex and correlate with systemic inflammation. Comparison of circulating LCN-2 levels in PDAC patients with and without cachexia **(A)**. Comparison of circulating LCN-2 levels between male and female PDAC patients **(B)**. CRP/Albumin ratio in PDAC patients within the indicated study groups (n=44) **(C)**. Relationship between circulating LCN-2 levels and CRP/Albumin ratio in PDAC patients (n=44) **(D)**. Scatter plots **(A–C)** show the median + IQR and individual data points in each group. Mann-Whitney U test for analysis of two groups; Kruskal-Wallis test followed by Dunn’s post-testing for analysis of multiple groups. Spearman’s rank correlation coefficient (*r_s_*) was used to test for the relationship between variables. Significant differences among the groups are signified by asterisks (*p < 0.05, **p < 0.01).

To assess whether LCN-2 levels are altered in patients with cancer cachexia, we determined circulating LCN-2 concentrations by ELISA. Whereas higher LCN-2 levels were observed in cachectic patients (median 26.7 (19.7-34.8) ng/mL) as compared to non-cachectic patients (median 24.8 (16.6-29.4) ng/mL), the difference was not significant (*p*=0.141, **Figure 2A**). In line with other studies (25–27), circulating LCN-2 levels showed a sex-specific difference, being higher in males than in females (median 27.8 (23.8-37.8) ng/mL *vs*. 21.6 (15.9-29.0) ng/mL, *p*=0.002, **Figure 2B**).

Since systemic inflammation is a hallmark of cancer cachexia, and because LCN-2 release is associated with inflammation, we determined the degree of systemic inflammation as expressed by the CRP to albumin ratio in the study groups. As expected, a significantly higher CRP/Albumin ratio was observed in cachectic PDAC patients with high LCN-2 levels as compared with patients without cachexia (2.3 (1.3-6.0) *vs*. 1.0 (0.4-1.4), *p*=0.041, **Figure 2C**). Furthermore, circulating LCN-2 levels correlated positively with the CRP/Albumin ratio (*r_s_* =0.30, *p*=0.047, **Figure 2D**).

### Systemic lipocalin 2 levels correlate with levels of neutrophil activation markers

**Figure 3:**
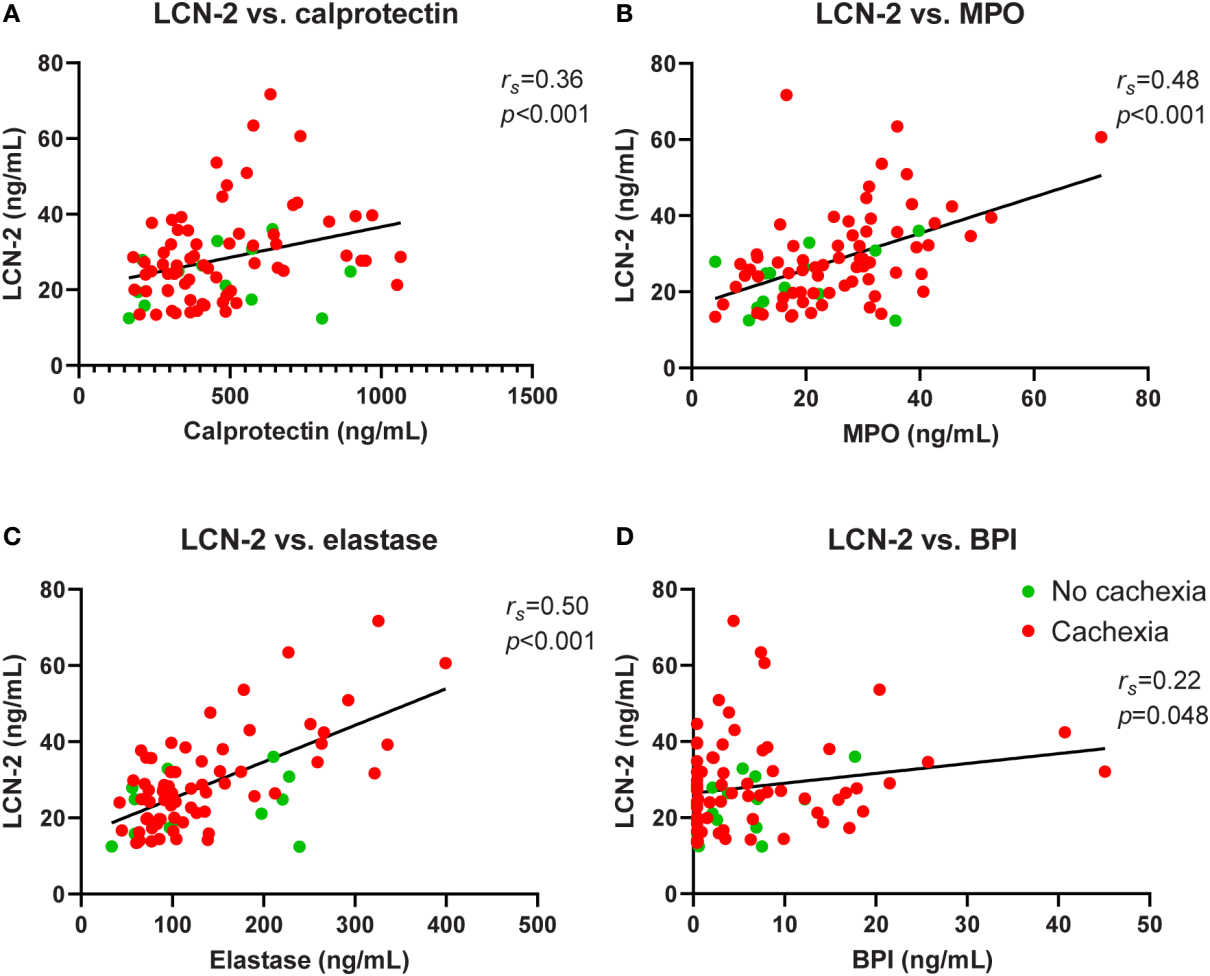
Correlation analysis of circulating LCN-2 and neutrophil activation markers in PDAC patients. Systemic levels of LCN-2 were positively correlated with levels of calprotectin **(A)**, MPO **(B)**, elastase **(C)**, and BPI **(D)**. Spearman’s rank correlation coefficient (*r_s_*) and level of significance are indicated in the respective plots. N=81.

LCN-2 can be produced by many different cell types, including cells relevant to cachexia such as adipocytes and hepatocytes (28, 29). However, it was previously shown that in experimental cachexia in mice, neutrophils were the main source of LCN-2 (20). To investigate the contribution of neutrophils to the systemic LCN-2 pool in pancreatic cancer patients with and without cachexia, we quantified circulating levels of reliable neutrophil activation markers calprotectin, MPO, elastase, and BPI in relation to levels of LCN-2. We observed consistent significant positive correlations between the concentrations of LCN-2 and all tested neutrophil activation markers (calprotectin: *r_s_* =0.36, *p*<0.001; **Figure 3A**; MPO: *r_s_* =0.48, *p*<0.001; **Figure 3B**; neutrophil elastase: *r_s_* =0.50, *p*<0.001; **Figure 3C**; BPI: *r_s_* =0.22, *p*=0.048; **Figure 3D**). Moreover, levels of calprotectin, MPO, elastase, and BPI were also strongly positively correlated to each other (**Figure 1**).

**Figure 4:**
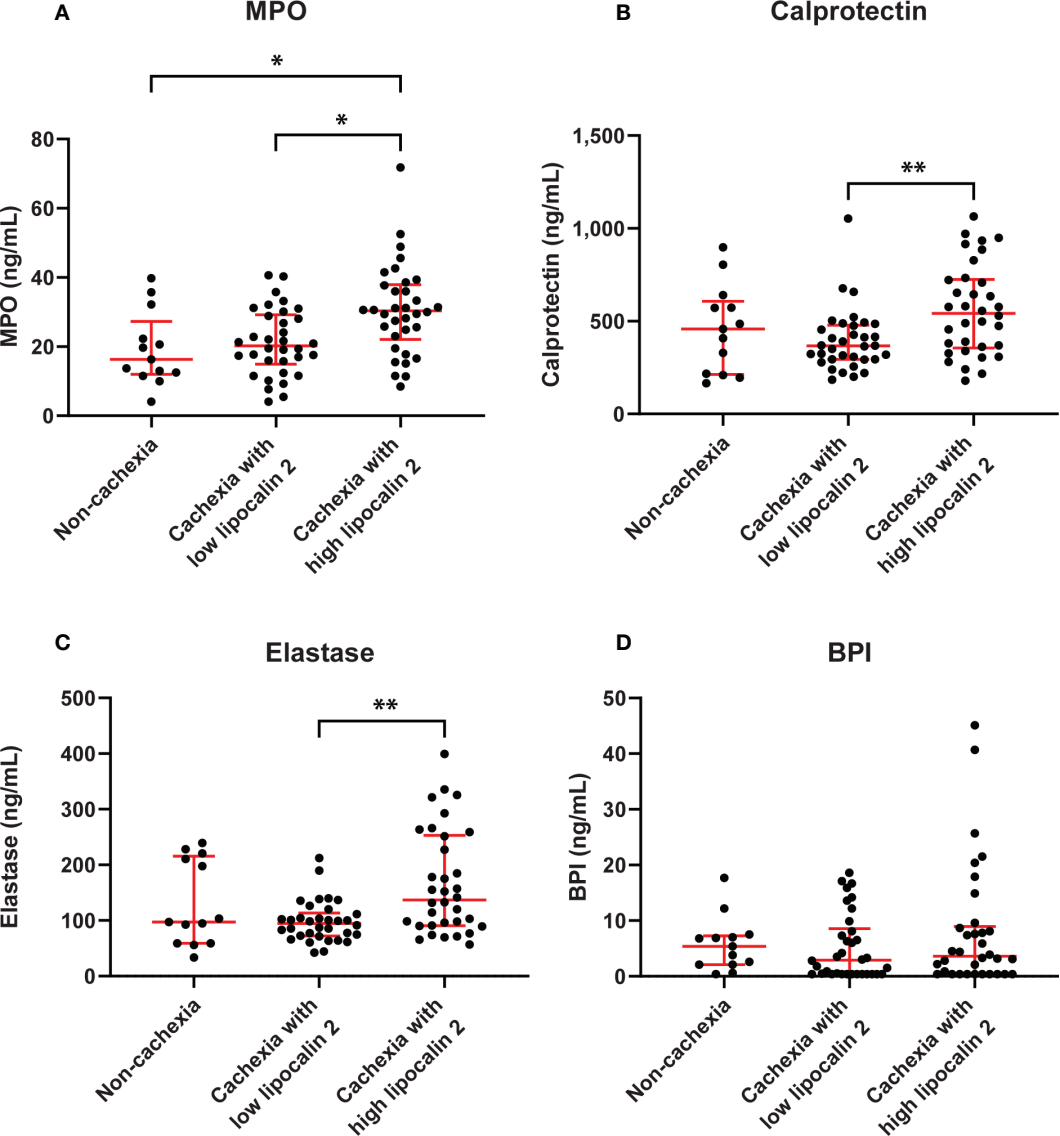
Circulating levels of neutrophil activation markers in PDAC patients without cachexia and in cachectic patients with high or low LCN-2 levels. Comparison of systemic levels of calprotectin **(A)**, MPO **(B)**, elastase **(C)**, and BPI **(D)** in PDAC patients without cachexia and in cachectic patients with high or low LCN-2 levels. Scatter plots show the median + IQR and individual data points in each group. For statistical analysis, the Kruskal-Wallis test followed by Dunn’s multiple comparisons test was used. Significant differences among the groups are signified by asterisks (*p < 0.05, **p < 0.01).

In addition, when comparing cachectic patients with high versus low levels of LCN-2, the median MPO levels of cachectic patients in the high LCN-2 group were significantly higher than those of cachectic patients with low LCN-2 or those found in patients without cachexia (median 30.3 (22.1-37.9) ng/mL *vs*. 20.2 (15.0-29.2) ng/mL, *p*=0.011; *vs*. 16.3 (12.0-27.5) ng/mL, *p*=0.021, respectively) (**Figure 4A**). Similarly, cachectic patients with high LCN-2 levels had significantly higher concentrations of calprotectin and elastase than cachectic patients with low LCN-2 levels (calprotectin: median 542.3 (355.8-724.9) ng/mL *vs*. 366.5 (294.5-478.5) ng/mL, *p*=0.009, elastase: 137.1 (90.8-253.2) ng/mL *vs*. 95.0 (72.2-113.6) ng/mL, *p*=0.006) (**Figures 4B, C**). However, no significant differences in calprotectin and elastase levels were observed between patients without cachexia and patients with cachexia with either high or low LCN-2 levels (**Figures 4B, C**). For BPI, no significant differences were observed between cachectic patients with high LCN-2 (median 3.6 (0.4-8.9) ng/mL) and patients without cachexia (5.4 (2.1-7.3) ng/mL, *p*=0.584) or cachectic patients with low LCN-2 levels (2.9 (0.4-8.6) ng/mL, *p*=0.931) (**Figure 4D**). Taken together, these data strongly indicate that LCN-2 levels in PDAC patients are associated with neutrophil activation.

### Circulating LCN-2 does not correlate with cachexia features

**Figure 5:**
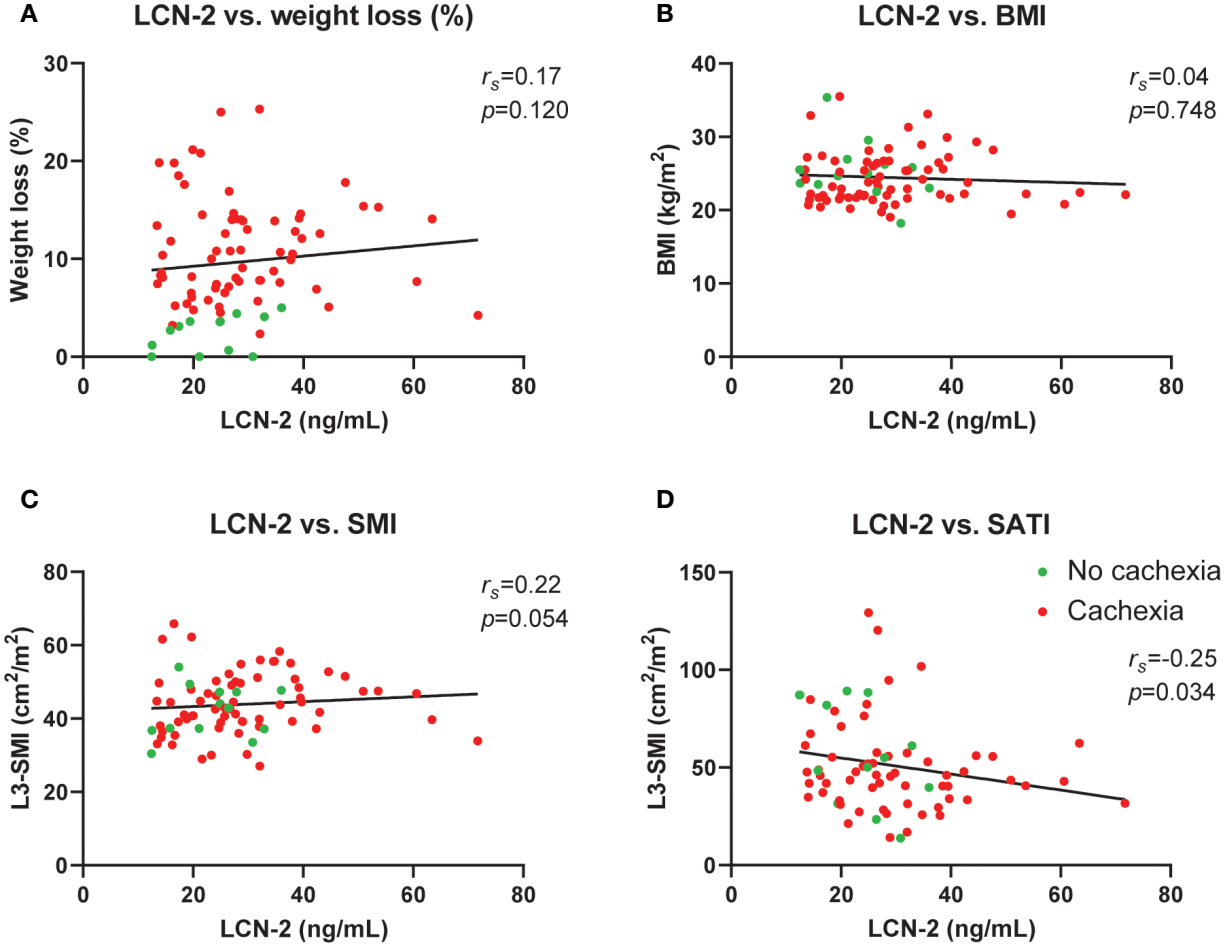
Circulating LCN-2 levels do not correlate with cachexia features in pancreatic cancer patients. Correlation analysis between circulating LCN-2 levels and cachexia features weight loss (%) **(A)**, body mass index **(B)**, skeletal muscle index (n=80) **(C)**, as well as subcutaneous fat index (n=72) **(D)**. Spearman’s rank correlation coefficient (*r_s_*) was used for the relationship between variables.

To investigate whether circulating LCN-2 levels are associated with specific cachexia features in PDAC patients, we performed correlation analyses. As shown in **Figures 5A–D**, no correlations between circulating LCN-2 levels and cachexia features such as weight loss (%) (*r_s_* =0.17, *p*=0.120, **Figure 5A**), body mass index (*r_s_* =0.04, *p*=0.748, **Figure 5B**), or skeletal muscle index (*r_s_* =0.22, p=0.054, **Figure 5C**) were observed. However, a negative correlation between plasma LCN-2 and subcutaneous fat index (SATI) was found (*r_s_* =-0.25, *p*= 0.034) (**Figure 5D**).

### Neutrophil activation is associated with complement system activation in PDAC patients

**Figure 6:**
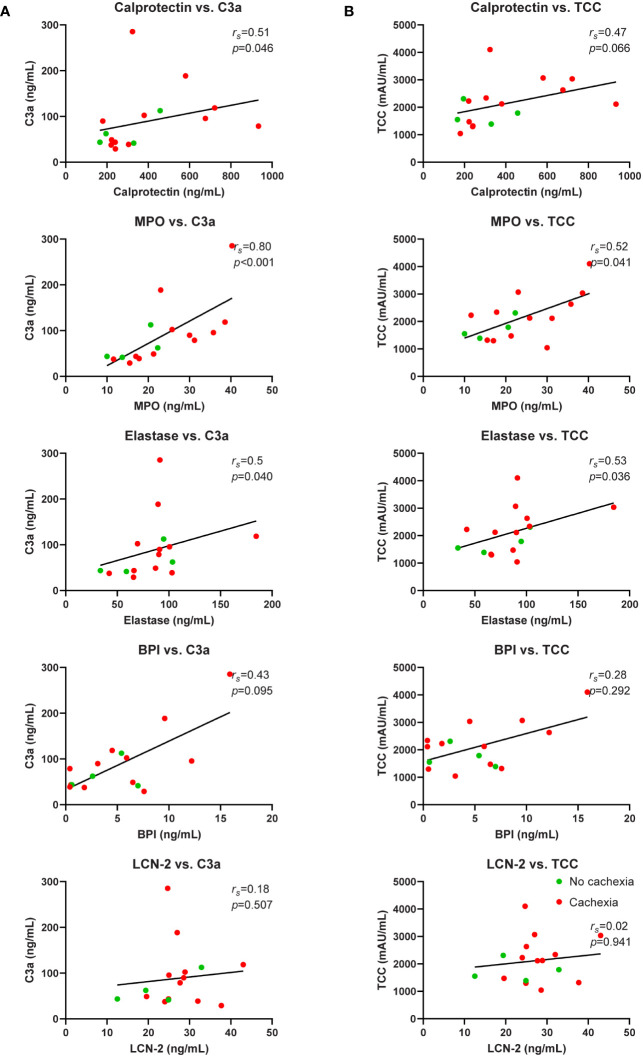
Correlation analysis of neutrophil activation markers and complement system activation markers. Relationship between systemic levels of C3a and calprotectin, MPO, elastase, BPI, and LCN-2 **(A)**. Relationship between systemic levels of TCC and calprotectin, MPO, elastase, BPI, and LCN-2 **(B)**. n=16 for each graph. Spearman’s rank correlation coefficient (*r_s_*) and level of significance are indicated in the respective plots.

Next, we focused on potential causes of the neutrophil activation. Since it is well-known that complement factors promote neutrophil activation (30) and because we previously reported complement system activation in patients with cancer cachexia (31), we investigated whether neutrophil activation was associated with complement system activation in a subgroup of patients in the current cohort that overlapped with the cohort reported on in (31) (n=16). Patient characteristics of this subgroup are shown in **Supplementary Table S1**. Interestingly, both C3a, a cleavage product of the central complement C3 component, and terminal complement complex (TCC), an end product of complement activation, were strongly positively correlated with the studied neutrophil activation markers calprotectin (C3a: *r_s_* =0.51, *p*=0.046; TCC: *r_s_* =0.47, *p*=0.066; **Figures 6A, B**), MPO (C3a: *r_s_* =0.80, *p*=<0.001; TCC: *r_s_* =0.52, *p*=0.041; **Figures 6A, B**), elastase (C3a: *r_s_* =0.52, *p*=0.040; TCC: *r_s_* =0.53, *p*=0.036; **Figures 6A, B**), and BPI (C3a: *r_s_* =0.43, *p*=0.095; TCC: *r_s_* =0.28, *p*=0.292; **Figures 6A, B**). This suggests that complement activation may contribute to neutrophil activation in patients with pancreatic cancer.

### LCN-2 levels in severely malnourished patients with pancreatic cancer

**Figure 7:**
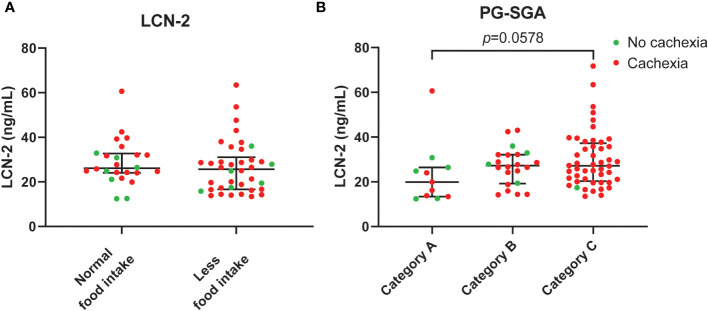
Circulating LCN-2 levels in PDAC patients according to food intake and nutritional status. Comparison of systemic levels of LCN-2 in PDAC patients with normal versus reduced food intake **(A)**. LCN-2 levels in plasma from well-nourished (category A), moderately malnourished (category B), and severely malnourished (category C) PDAC patients **(B)**. Scatter plots showing the median + IQR and individual data points in each group. For statistical analysis, the Mann–Whitney U test was used for two groups and the Kruskal-Wallis test followed by Dunn’s post-testing for analysis of multiple groups.

Given that administration of LCN-2 has been shown to suppress appetite in mouse models of pancreatic cancer cachexia (20), we next examined the link between LCN-2 levels and the nutritional status of patients using the validated PG-SGA questionnaire, which contains questions about food intake. Whereas patients with cachexia had a higher prevalence of poor appetite than non-cachectic patients, the difference was not significant (65.3% (32/49) *vs*. 46.2% (6/13), **Table 1**, *p*=0.448). Moreover, no significant difference was observed between PDAC patients with normal food intake and PDAC patients with less food intake in terms of circulating LCN-2 (median 26.1 (24.1-32.7) ng/mL *vs*. 25.7 (16.7-31.0) ng/mL, *p*=0.320, **Figure 7A**). However, we found that LCN-2 levels tended to be higher in patients with poor nutritional status (PG-SGA category A *vs*. category B *vs*. category C, median 19.9 (13.4-26.4) ng/mL *vs*. 27.2 (19.3-32.1) ng/mL *vs*. 27.2 (20.3-37.2) ng/mL, *p*=0.058, **Figure 7B**).

## Discussion

It was previously reported that increases in LCN-2 levels in pancreatic cancer patients correlate with loss of fat and muscle, two key features of cachexia (20). Based on relatively weak correlations to neutrophil abundance and the neutrophil/lymphocyte ratio, the LCN-2 elevations were attributed to neutrophil expansion (20). The current study provides several additional lines of evidence for a contribution of neutrophil activation to the elevated LCN-2 levels in patients with pancreatic cancer. We showed strong correlations between circulating levels of LCN-2 and the degree of systemic inflammation (CRP/albumin ratio) as well as a set of four different neutrophil activation markers, and demonstrated that cachectic patients with high systemic LCN-2 levels have significantly higher levels of the neutrophil activation markers calprotectin, MPO, and elastase than patients with low LCN-2 levels. Furthermore, consistent correlations between these neutrophil activation markers and activated complement factors C3a and TCC were observed in these patients, suggesting that systemic complement activation may contribute to neutrophil activation in pancreatic cancer. Of note, although circulating LCN-2 levels were not related to cachexia and food intake, higher LCN-2 levels were associated with worse nutritional status of patients, providing some support for the concept that LCN-2 contributes to cancer cachexia by suppressing patients’ appetite. Taken together, these results suggest that LCN-2 levels in cachectic patients with pancreatic cancer are related to neutrophil activation and complement activation.

LCN-2 is a polypeptide released by several cell types including adipocytes, hepatocytes, epithelial cells, and neutrophils. Elevated circulating LCN-2 has been found in many types of cancer and promotes malignant development in cancer patients (18, 32). The functional roles of LCN-2 include regulating body fat mass and lipid metabolism as well as immune responses to inflammatory stimuli. As a biomarker of inflammation, LCN-2 has been associated with chronic inflammatory disorders such as inflammatory bowel disease, obesity, and pancreatic cancer (18, 33, 34). In line with this, we found a positive correlation between circulating LCN-2 levels and systemic inflammation. However, LCN-2 levels were not associated with cachexia status of the patients. Of note, the mean plasma concentrations of LCN-2 in our cohort were considerably lower than the serum levels reported by others (20, 35). It is well-known that neutrophils become rapidly activated by many common preparation methods (36). In particular, activation of neutrophils during serum preparation is common, and it is therefore advisable to analyze neutrophil products in plasma instead of serum. Moreover, delays in blood processing have been shown to be associated with neutrophil death leading to artefactual increases in neutrophil products (37). To avoid neutrophil activation and death during plasma preparation, we applied careful centrifugation using freshly obtained blood, which is unlikely to be performed in retrospective studies where blood was routinely collected and processed at clinical chemistry departments.

Recently, emerging evidence revealed that LCN-2 suppresses appetite in mice. For example, Mosialou and colleagues demonstrated that LCN-2 suppresses food intake in mice by crossing the blood-brain barrier and binding to its receptor MC4R in the hypothalamic paraventricular nucleus (19). Similar appetite suppression by LCN-2 was observed in primates who received daily administration of recombinant human LCN-2 which resulted in a 21% decrease in food intake (38). In the context of cancer cachexia, a more recent study showed that administration of LCN-2 to mice reduced food intake and decreased body weight, while deletion of LCN-2 restored appetite in a mouse model of pancreatic cancer cachexia (20). To explore the relevance of this finding in human pancreatic cancer cachexia, we compared LCN-2 levels between PDAC patients with normal or reduced food intake and investigated the relationship between circulating LCN-2 and several features of cachexia including weight loss, body composition, and nutritional status. While LCN-2 is able to suppress appetite in mice with pancreatic cancer cachexia, we did not observe a relationship between food intake and LCN-2 levels in our patient cohort. Moreover, circulating LCN-2 levels did not correlate with weight loss and body mass index, although a significant negative association between circulating LCN-2 and the subcutaneous adipose tissue volume was observed. Also, LCN-2 levels were higher in patients that were malnourished according to the PG-SGA. Thus, although our data do not provide evidence for a direct link between LCN-2 and appetite in the context of human pancreatic cancer cachexia, LCN-2 may still indirectly affect cachexia-related nutritional factors.

Previously, we reported complement system activation in pancreatic cancer patients with cachexia (31). To gain an understanding of the potential relationship between neutrophil activation and complement activation in PDAC patients with cachexia, we performed correlation analyses. Intriguingly, we found strong correlations between all neutrophil activation markers studied and the central complement system activation markers C3a and TCC. A previous *in vitro* study showed that neutrophils can activate the alternative pathway of complement and release C5 fragments that further enhance neutrophil activation (30), which is in line with our current observations. Furthermore, several studies have shown that the treatment of human neutrophils with C3a leads to neutrophil degranulation, aggregation, and chemotaxis (39, 40). Thus, complement and neutrophil activation in these patients may result from a positive feedback loop.

In obesity and type 2 diabetes, neutrophilic inflammation has been shown to be involved in the development of insulin resistance and other metabolic aberrations (41, 42). In line with this, we found a correlation between SMRA, which reflects the extent of lipid accumulation in skeletal muscle, and levels of the neutrophil activation markers elastase and BPI (see **Figure 1**), which could suggest that neutrophil activation also promotes inflammation in skeletal muscle tissue of pancreatic cancer patients leading to insulin resistance and ectopic fat accumulation. In addition, we could corroborate the previously described differences in LCN-2 levels between males and females (25–27), with higher levels in males. It would be interesting to explore if this contributes to recently reported sex differences on cancer cachexia progression (43, 44), also given that we identified a correlation between LCN-2 and SATI, which is higher in females.

Certain limitations of this first clinical study on circulating LCN-2 levels in association with neutrophil activation in pancreatic cancer patients should be acknowledged. First, the study population was relatively small, and our results should be validated in a larger patient cohort. Second, the applied cut-off value for LCN-2 was based on the median value in cachectic patients which should be optimized by generating ROC curves in future large cohort studies. Third, although sarcopenia (defined by low SMI) is usually strongly associated with cachexia, cachectic patients in our study did not have a lower SMI than non-cachectic patients. Since self-reported unintentional weight loss of >5% is the central diagnostic criterion for cachexia, this “subjective” value could obscure actual differences in the SMI of each group. Fourth, given the reported role of LCN-2 in regulating appetite, a more detailed and objective assessment of nutritional intake using validated questionnaires would be desirable in follow up studies. Finally, although a strong positive correlation between circulating LCN-2 and neutrophil activation markers was observed which is in line with activated neutrophils as the main source of LCN-2, we cannot exclude the contribution of bone-derived LCN-2 to circulating levels in the patients studied.

In conclusion, the present study shows that circulating LCN-2 is associated with neutrophil activation in pancreatic cancer patients, irrespective of their cachexia status. Generalized activation of the innate immune system seems to contribute to the production of circulating LCN-2 as indicated by the correlations between neutrophil activation markers and activated complement components. Follow-up studies investigating the potential of LCN-2 as a therapeutic target in cancer cachexia are warranted given the association between its levels and the nutritional status of PDAC patients.

## Data availability statement

The raw data supporting the conclusions of this article will be made available by the authors, without undue reservation.

## Ethics statement

The studies involving human participants were reviewed and approved by the Medical Ethical Board of the MUMC+. The patients/participants provided their written informed consent to participate in this study.

## Author contributions

Conceptualization: SR. Methodology: SR and MD. Formal analysis: MD and AB. Resources: SO and SR. Data curation: SR. Writing—original draft preparation: MD and SR. Writing—review and editing: MA, GK, KL, UN, GW, FS, AB, and SO. Supervision: SO and SR. All authors contributed to the article and approved the submitted version.
