# Supplementary material for: Lipocalin-2 and neutrophil activation in pancreatic cancer cachexia
Source: Front Immunol. 2023 Mar 15;14:1159411. doi: 10.3389/fimmu.2023.1159411 (PMC10057111; doi:10.3389/fimmu.2023.1159411)
Supplement: Supplementary file 1 [file Table_1.docx]

**Supplementary tables**

Supplementary Table S1. Patient characteristics

|  | **Overall** | **No cachexia** | **Cachexia** | ***p*-value** |
| --- | --- | --- | --- | --- |
| *n* | 16 | 4 | 12 |  |
| Age (year) | 71.0 (59.0, 75.0) | 64.0 (54.5, 74.2) | 71.0 (63.0, 75.0) | 0.715 |
| BMI (kg/m^2^) | 24.2 (22.6, 26.5) | 25.2 (24.4, 26.8) | 23.8 (21.8, 26.5) | 0.275 |
| Weight loss (%) | 7.4 (4.4, 11.3) | 3.6 (3.0, 3.7) | 9.5 (6.9, 12.9)* | **0.004** |
| SMRA (HU) | 36.9 (29.8, 40.1) | 38.8 (34.9, 41.2) | 35.7 (29.8, 39.3) | 0.396 |
| L3-SMI (cm^2^/m^2^) | 42.2 (39.2, 48.3) | 40.6 (37.1, 45.4) | 42.2 (39.8, 48.3) | 0.467 |
| L3-VATI (cm^2^/m^2^) | 54.9 (30.6, 74.7) | 39.0 (30.5, 47.0) | 64.0 (32.2, 78.7) | 0.182 |
| L3-SATI (cm^2^/m^2^) | 51.9 (32.5, 71.8) | 74.2 (53.8, 87.4) | 50.6 (31.4, 56.7) | 0.192 |
| CRP/Albumin | 0.9 (0.4, 2.5) | 0.5 (0.4, 0.9) | 1.0 (0.6, 6.0) | 0.404 |
| LCN-2 (ng/mL) | 26.0 (24.5, 29.7) | 22.1 (17.7, 26.9) | 27.4 (24.8, 29.7) | 0.203 |
| Calprotectin (ng/mL) | 314.2 (221.9, 488.4) | 262.4 (187.9, 361.5) | 314.2 (235.2, 605.1) | 0.332 |
| MPO (ng/mL) | 21.8 (16.6, 30.3) | 17.1 (12.8, 21.0) | 24.4 (17.6, 32.3) | 0.090 |
| Elastase (ng/mL) | 89.9 (66.1, 96.3) | 76.8 (52.5, 97.1) | 89.9 (68.8, 93.6) | 0.716 |
| BPI (ng/mL) | 5.0 (1.5, 7.2) | 4.0 (2.1, 5.8) | 5.2 (1.5, 8.1) | 0.716 |
| C3a (ng/mL) | 70.6 (43.0, 104.9) | 53.0 (43.1, 74.8) | 84.4 (42.4, 106.4) | 0.716 |
| TCC (mAU/mL) | 2117.2 (1452.2, 2412.0) | 1670.6 (1511.9, 1919.4) | 2173.1 (1434.2, 2731.6) | 0.467 |

The data are presented as median + IQR. Groups were compared using the Mann–Whitney U test. * Significant difference in comparison to the no cachexia group. BMI: body mass index; HU: Hounsfield unit; L3-IMAT: L3-intermuscular adipose tissue; SMRA: skeletal muscle radiation attenuation; L3-SMI: L3-muscle index; L3-VATI: L3-visceral adipose tissue index; L3-SATI: L3-subcutaneous adipose tissue index; CRP: C-reactive protein; LCN-2: lipocalin 2; MPO: myeloperoxidase: BPI: bactericidal permeability increasing protein (BPI); TCC: terminal complement complex.
